# Gut microbiota severely hampers the efficacy of NAD-lowering therapy in leukemia

**DOI:** 10.1038/s41419-022-04763-3

**Published:** 2022-04-08

**Authors:** Oussama ElMokh, Saki Matsumoto, Paulina Biniecka, Axel Bellotti, Karin Schaeuble, Francesco Piacente, Hector Gallart-Ayala, Julijana Ivanisevic, Ivan Stamenkovic, Alessio Nencioni, Aimable Nahimana, Michel A. Duchosal

**Affiliations:** 1 Central Laboratory of Hematology, Department of Medical Laboratory and Pathology, Lausanne University Hospital and University of Lausanne, 27-sud, Rue du Bugnon, CH-1011 Lausanne, Switzerland; 2 Department of Oncology UNIL CHUV, University of Lausanne, 1066 Epalinges, Switzerland; 3 Department of Internal Medicine, University of Genoa, 16132 Genoa, Italy; 4 Metabolomics Unit, Faculty of Biology and Medicine, University of Lausanne, 1005 Lausanne, Switzerland; 5 Department of Formation and Research, Lausanne University Hospital and University of Lausanne, Lausanne, CH-1011 Switzerland; 6 Service of Hematology, Department of Oncology, Lausanne University Hospital and University of Lausanne, 46, Rue Bugnon, 1011 Lausanne, Switzerland

**Keywords:** Drug development, Cancer metabolism

## Abstract

Most cancer cells have high need for nicotinamide adenine dinucleotide (NAD^+^) to sustain their survival. This led to the development of inhibitors of nicotinamide (NAM) phosphoribosyltransferase (NAMPT), the rate-limiting NAD^+^ biosynthesis enzyme from NAM. Such inhibitors kill cancer cells in preclinical studies but failed in clinical ones. To identify parameters that could negatively affect the therapeutic efficacy of NAMPT inhibitors and propose therapeutic strategies to circumvent such failure, we performed metabolomics analyses in tumor environment and explored the effect of the interaction between microbiota and cancer cells. Here we show that tumor environment enriched in vitamin B3 (NAM) or nicotinic acid (NA) significantly lowers the anti-tumor efficacy of APO866, a prototypic NAMPT inhibitor. Additionally, bacteria (from the gut, or in the medium) can convert NAM into NA and thus fuel an alternative NAD synthesis pathway through NA. This leads to the rescue from NAD depletion, prevents reactive oxygen species production, preserves mitochondrial integrity, blunts ATP depletion, and protects cancer cells from death.

Our data in an in vivo preclinical model reveal that antibiotic therapy down-modulating gut microbiota can restore the anti-cancer efficacy of APO866. Alternatively, NAphosphoribosyltransferase inhibition may restore anti-cancer activity of NAMPT inhibitors in the presence of gut microbiota and of NAM in the diet.

## Graphical abstract

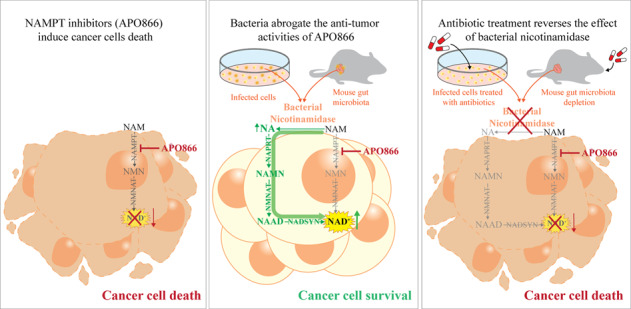

## Introduction

Compared to normal cells, most cancer cells have a high demand for nutrients and essential cofactors such as glucose, glutamine, and nicotinamide adenine dinucleotide (NAD^+^), which sustain cancer cell proliferation and survival [1–3]. Tumor cells are expected to be more vulnerable to NAD^+^ depletion than normal cells [4]. This notion led to the development of NAD^+^ synthesis inhibitors for the clinical treatment of cancer [4–6].

Mammalian cells synthesize NAD^+^ mainly through the salvage pathway utilizing nicotinamide (NAM) as a substrate, but also from other precursors that include tryptophan (via *de novo* pathway), nicotinic acid (NA, through the Preiss-Handler pathway), ribosylated NAM (NR) or NA (NAR), as well as the reduced form of NR (NRH) [7–14]. NAMPT is the rate-limiting enzyme that catalyzes the phosphoribosylation of NAM to produce nicotinamide mononucleotide (NMN). Despite the therapeutic efficacy of NAMPT inhibitors reported in several preclinical studies of solid and blood cancers [5, 15–21], the most promising agents (APO866 and GMX-1777) failed in clinical studies [22, 23], suggesting that alternative NAD^+^ production routes may be active in humans. Shats et al. [24] recently reported that intestinal bacteria boost NAD^+^ production in mammalian tissues through the activity of their enzyme, nicotinamidase (NMASE or PncA), which bypasses NAMPT inhibitors activity, and counteracts the anti-tumor effects of NAMPT inhibitor in a colon carcinoma cell line. Whether and how the microbiota may alter the anti-lymphoma/leukemia properties of NAMPT inhibitors has not been addressed.

In humans, the concentration of vitamin B3 (NA, NAM and related riboside derivatives) in the blood is very variable, with values ranging from 35 to 1487 nM and it is influenced by the type of diet and by the feeding status [25]. The level of NAD^+^ precursors may considerably affect the anti-tumor efficiency of NAMPT inhibitors. These inhibitors induce individually variable levels of side effects, including retinopathy and thrombocythemia [22, 26]. In this study, we investigated the effect of tumor metabolic environment and the influence of the interaction between NA/NAM and the gut microbiota on the anti-leukemic activity of APO866. We show that tumor environments that are enriched in NA/NAM from the diet, markedly affect APO866 therapeutic efficacy. Furthermore, we show that when the tumor environment is specifically enriched in NAM, the anti-leukemic effect of APO866 is modulated by the levels of intestinal bacteria.

## Materials and methods

### Cell lines, primary cells and culture conditions

A panel of 21 hematopoietic cancer cell lines and primary cells from patients (listed in Table S1 and S2), was evaluated. *Escherichia coli* and *Saccharomyces cerevisiae* strains were kindly supplied by Dr Philippe Hauser (Institute of Microbiology, Lausanne University Hospital, Lausanne, Switzerland). All cells were cultured in RPMI (Invitrogen AG, 61870-01) supplemented with 10% heat inactivated fetal calf serum (Amimed, 2-01F30-I) and 1% penicillin/streptomycin at 37 °C (Amimed, 4-01F00-H) in a humidified atmosphere of 95% air and 5% CO2. To eliminate mycoplasma from cell culture, mycoplasma-infected leukemic cells, were cultured in the medium (as mentioned above), supplemented with BM-cyclin (Roche, Mannheim, Germany; Cat. No. 10799050001) according to the manufacturer’s instructions.

### Cell death characterization

Uninfected or bacteria-infected leukemic cells were cultured without or with APO866 (as indicated in each figure) in presence or absence of NAD^+^ precursors. APO866-induced cell death was determined using ANNEXIN-V (ANN; eBioscience, BMS306FI/300) and 7 aminoactinomycin D (7AAD; Immunotech, A07704) stainings as described by the manufacturer and analyzed using flow cytometry. Dead cells were identified as ANN+ and/or 7AAD+.

### Genome editing by CRISPR method

Single guide RNAs targeting the early exon (exon number 2) of PARP1 were chosen in the sgRNA library [27]. LentiCRISPR plasmid specific for NAPRT gene was created according to the provided instructions. Oligonucleotides were designed as follow: Forward 5’- CACCGCCCACCTGGCGTAGCTGACC-3’; Reverse 3’- AAACGGTCAGCTACGCCAGGTGGGC-5’. Oligonucleotides were synthetized, then phosphorylated and annealed to form oligo complex. LentiCRISPR vector was BsmBI digested and dephosphorylated. Linearized vector was purified, and gel extracted and ligated to oligo complex. The lentiCRISPR vector containing the sgRNA was then used for virus production. Cells were infected and selected with the appropriate dose of puromycin (1 µg/ml). Clone isolation was performed by limiting dilution in 96 well-plates.

### TA cloning

TA cloning kit (Life technologies, K202020) was used according to manufacturer’s instructions to sequence DNA fragment containing the region where Cas9 was guided by a sgRNA.

### Immunoblotting

Protein samples were harvested in lysis buffer containing 20 mM HEPES, pH 7.4, 10 mM NaCl, 3 mM MgCl_2_, 2.5 mM EGTA, 0.1 mM dithiothreitol, 50 mM NaF, 1 mM Na_3_VO_4_. A protease inhibitor cocktail (Roche, 11873580001) was added. Lysates were sonicated and protein concentration was determined using a Bradford assay. Proteins (25–40 μg) were separated by SDS-PAGE on a 10% polyacrylamide gel and analyzed by immunoblotting. The mouse anti-NAPRT (#86634) and the rabbit anti-actin antibodies were purchased from Cell Signaling. After incubation with primary antibodies, the following secondary antibodies were applied: polyclonal goat anti-mouse or goat anti-rabbit IgG conjugated with IRDye 680 (LI-COR, B70920-02) or IRDye 800 (LI-COR, 926-32210). Protein bands were visualized using the Odyssey Infrared Imaging System (LI-COR). Full and uncropped western blots were uploaded as ‘Supplemental Material’.

### Quantification of NAD^+^ metabolites using LC-MS/MS

#### Sample extraction

Serum samples (25 µL) were extracted with 225 µl of ice-cold methanol containing stable isotope-labeled metabolites. Sample extracts were centrifuged (15 min, 14000 rpm at 4 °C). The supernatant was collected and evaporated to dryness in a vacuum concentrator (LabConco, Missouri, US). Then sample extracts were reconstituted in 75 µL of water prior to LC-MS/MS analysis.

#### LC-MS/MS method

Extracted samples were analyzed by Liquid Chromatography coupled with tandem mass spectrometry (LC - MS/MS) in positive electrospray ionization (ESI) mode. An Agilent 1290 Infinite (Agilent Technologies, Santa Clara, California, US) ultra-high performance liquid chromatography (UHPLC) system was interfaced with Agilent 6495 LC-MS QqQ system equipped with an Agilent Jet Stream ESI source. This LC-MS/MS was used for the quantification of the intermediates implicated in NAD^+^ *de novo synthesis* and *salvage* pathways.

The separation of NAD^+^ metabolites implicated in salvage and Preiss-Handler pathway was carried out using the Scherzo SMC18 (3 µm 2.0 mm × 150 mm) column (Imtakt, MZ-Analysentechnik, Mainz, Germany). The mobile phase was composed of *A* = 20 mM Ammonium Formate and 0.1% formic acid in H_2_O and B = Acetonitrile: Ammonium formate 20 mM and 0.1% formic acid (90:10, v/v). The gradient elution started at 100% A (0–2 min), reaching 100% B (2 min–12 min), then 100% B was held for 3 min and decreased to 100% A in 1 min following for an isocratic step at the initial conditions (16 –22 min). The flow rate was 200 μL/min, column temperature 30 °C and the sample injection volume was 2 μL. To avoid sample carry-over injection path was cleaned after each injection using a strong solvent (methanol 0.2% formic acid) and weak solvent (0.2% formic acid in water).

AJS ESI source conditions operating in positive mode were set as follows: dry gas temperature 290 °C, nebulizer 45 psi and flow 12 L/min, sheath gas temperature 350 °C and flow 12 L/min, nozzle voltage +500 V, and capillary voltage +4000 V. Dynamic Multiple Reaction Monitoring (DMRM) acquisition mode with a total cycle of 600 ms was used operating at the optimal collision energy for each metabolite transition.

#### Data processing

Data were processed using Mass Hunter Quantitative (Agilent). For absolute quantification, the calibration curve and the internal standard spike were used to determine the response factor. Linearity of the standard curves was evaluated using a 14-point range; in addition, peak area integration was manually curated and corrected where necessary. The concentrations of metabolites were corrected for the ratio of peak area between the analyte and the ISTD, to account for matrix effects.

### Stool collection, bacterial DNA detection and quantification

Bacterial DNA from collected mouse stools were extracted using the Power Fecal PRO kit (ref. 51804). The quantitative PCR was performed with 5 µL DNA, 200 nM of each primer Eubact_27F (AGAGTTTGATCMTGGCTCAG) and Eubact_244R (ACTGCTGCCTCCCGTAG) [28] and 10 µL iTaq Universal SYBR Green Supermix (BioRad, Switzerland, ref. 172–5122) as follows: start 95 °C for 5’, denaturation at 95 °C for 15” and hybridization at 60 °C for 1’ repeated for 40 cycles. The analyses were performed on the StepOne Plus. Ten-fold dilutions of a control plasmid prepared by RDBiotech (France) was used to calibrate the qPCR.

### Generation of intestinal microbiota-depleted mice and evaluation of therapeutic efficacy of APO866 in mouse xenograft with intact or depleted intestinal bacteria

Six- to 8-week female non-leaky C.B.-17 severe combined immune deficiency (SCID) mice (Iffa Credo, L’ Arbresle, France) were bred and housed in micro-isolator cages in a specific pathogen-free room within the animal facilities at the University Hospital of Lausanne. Animals were allowed to acclimatize to their new environment for 1 week prior to use. All animals were handled according to the respective institutional regulations after approval of the animal ethics committee of the University of Lausanne. Depletion of the intestinal microbiota was performed as described elsewhere [29] using an antibiotic cocktail of streptomycin (500 mg/kg, Sigma-Aldrich, Saint-Louis, USA; Cat. No. S9137), gentamicin (125 mg/kg, Sigma-Aldrich, Saint-Louis, USA; Cat. No. G1264), bacitracin (250 mg/kg, Sigma-Aldrich, Saint-Louis, USA; Cat. No. 11702) and ciprofloxacin (67.5 mg/kg, Sigma-Aldrich, Saint-Louis, USA; Cat. No. 17850) in NaCl 0.9% administered daily by oral gavage. Stools were routinely collected. The sample size was chosen on the basis of an adequate power using a student test (T-test), between 2 means (Control vs treated), based on data from our previous studies [30]. The accepted statistical significativity (alpha) was 0.05, with an obtained adequate power of 0.85.

The in vivo evaluation of APO866 was carried out using a xenograft model of ML-2 human AML. SCID mice with intact or depleted intestinal bacteria were fed with various diets, enriched without or with either NAM or NA, at least one week before being transplanted subcutaneously into the right flank with ML-2 cells (10^7^). Once the tumor reached a volume of 100–150 mm^3^, mice were randomly subdivided into untreated (control or vehicle) and APO866-treated groups. Mice were administered intraperitoneally with APO866 (15 mg/kg body weight) in 200 µL 0.9% saline, twice a day for 4 days, repeated weekly over 3 weeks. Control groups were treated similarly with saline solution. All animals were monitored daily for signs of illness and killed immediately if tumor size reached a diameter of 15 mm. It is noteworthy to mention that depletion of the intestinal microbiota was confirmed before mice were xenografted. The investigator was not blinded to the group allocation during the experiment.

### Statistical analysis

Data are expressed as mean plus or minus standard error of the mean (SEM) unless otherwise noted. Values between groups were compared using non-parametric test. The Kaplan-Meier method using long rank test was applied for the analyses of animal survival studies. GraphPad Prism version 8.3.0 (GraphPad Software, San Diego, CA) was used for statistical analysis. P values less than .05 were considered statistically significant.

## Results

### Bacteria abrogate the anti-tumor activities of APO866 in several hematopoietic malignant cells

**Fig. 1:**
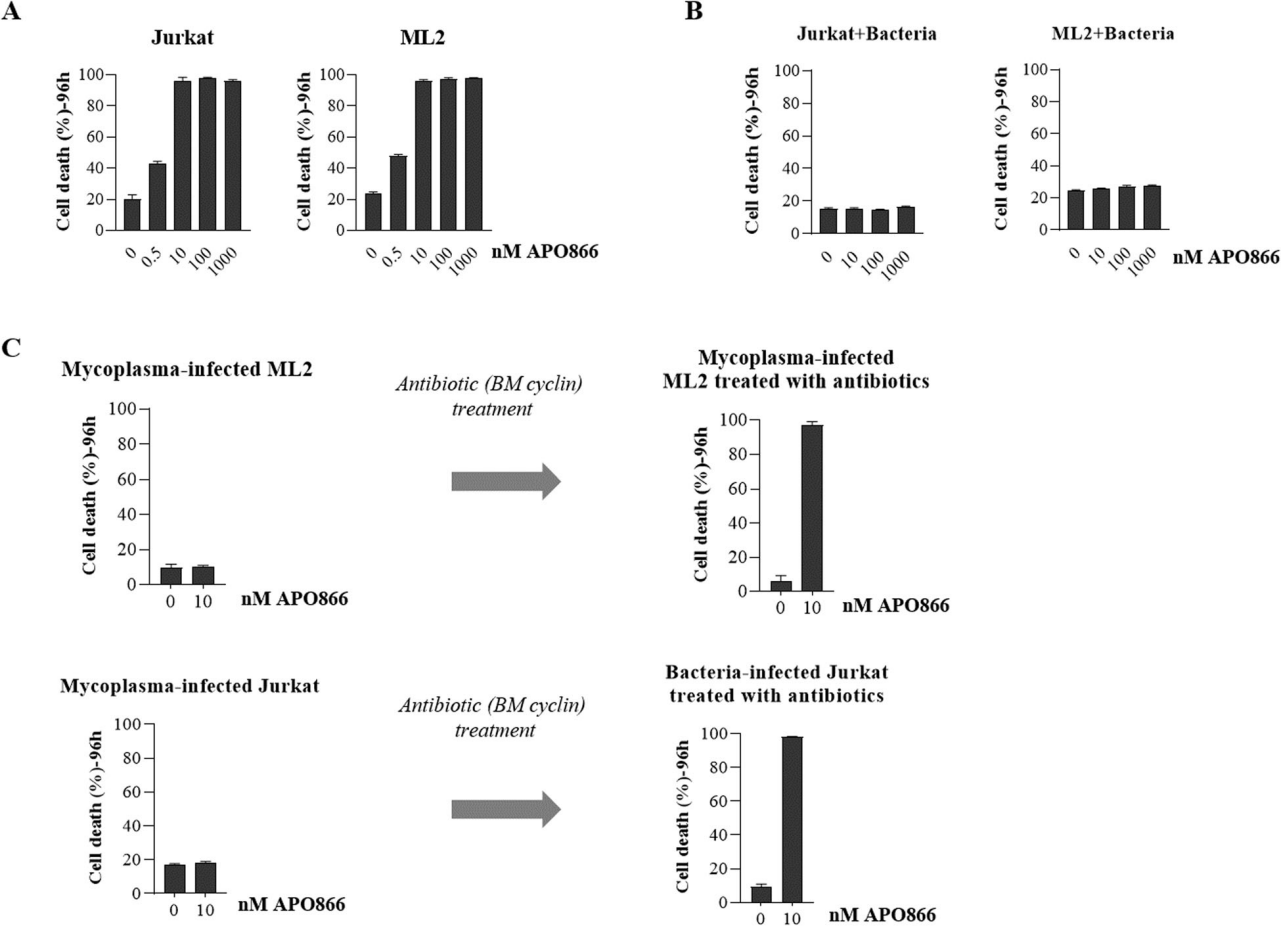
Bacteria protect leukemic cells from the anti-tumor activity of APO866 without affecting the drug uptake, the capacities to proliferate or to form colonies. Dose dependent analysis of cell death on two unrelated uninfected (**A**) and bacteria-infected (**B**) leukemic cells (Jurkat and ML2) exposed to APO866 for 96 h. Cell death was assessed using annexin and 7AAD stainings and analyzed by flow cytometry. Cells stained positively for either annexin or 7AAD alone or both were considered dead cells. **C** APO866 sensitivities of bacteria-infected ML2 and Jurkat cells, before and after antibiotic treatment. Data are mean ± SD, derived from at least three independent experiments.

**Fig. 2:**
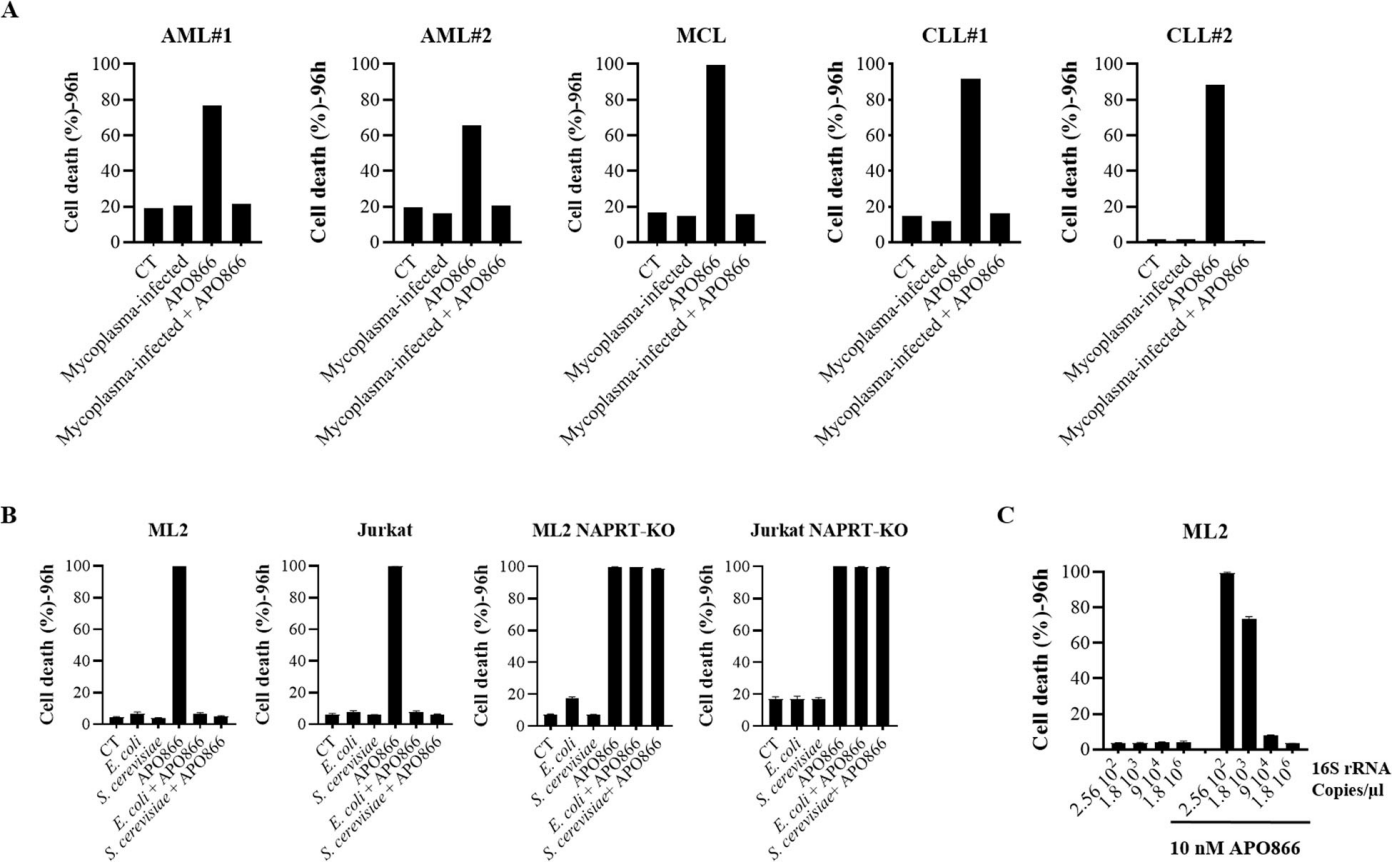
Bacteria abrogate the anti-leukemic/lymphoma effects of APO866 in primary cells from patients diagnosed with various hematological malignancies and in several leukemia/lymphoma cell lines. Cell death analysis on several uninfected and bacteria-infected primary leukemic cells (**A**) and ML-2 cells infected with different cell number of *E. coli* exposed to 10 nM APO866 was monitored as mentioned in Fig. 1. APO866 sensitivities of (**B**) ML-2 and Jurkat cells (both either WT or NAPRT-KO) infected with *E.coli* or *S.cerevisiae* and of (**C**) ML-2 cells exposed to different *E. coli* quantities (inocula). Cell death was assessed as described in Fig. 1. Data are mean ± SD, derived from at least three independent experiments.

To examine the effect of bacteria on the anti-leukemic effects of NAMPT inhibitors, we took advantage of cell lines that were infected or not with Mycoplasma. First, we sought to identify the Mycoplasma species infecting our cell cultures. Using a high-throughput detection and multiplex identification of cell contamination test [31], Table S3 shows that the cell cultures were infected with *Mycoplasma arginini*. Supernatant from Mycoplasma-contaminated cell cultures were used to infect other leukemic cell cultures (dilution factor: 100–200). Next, two unrelated uninfected and Mycoplasma-infected leukemia cell lines were incubated with various concentrations of APO866 (0–1000 nM) for 96 h and subsequently double stained using annexin V/7AAD to monitor cell death. As shown in Fig. 1A, APO866 effectively killed malignant hematopoietic cells: specifically, 10 nM APO866 was sufficient to kill 100% of the leukemic cells, which is in line with our previous studies [30, 32, 33]. This dose was therefore chosen for subsequent experiments. In contrast, the presence of Mycoplasma in cell culture fully abolished the anti-leukemia effects of APO866 (Fig. 1B). Of note, one can observe that Mycoplasma contamination per se did not affect neither drug uptake (Fig. S1A), nor cell viability (Fig. S1B), whereas it abrogated the capacity of APO866 to inhibit cell proliferation (Fig. S1C) and to block clonogenicity (Fig. S1D). Mycoplasma-infected leukemia cells were re-sensitized to APO866 after bacterial elimination with BM cyclin antibiotic treatments (Fig. 1C), thus confirming that bacteria were responsible for the lack of APO866 anti-tumor effect. It is noteworthy to mention that BM cyclin treatment eliminated Mycoplasma from infected leukemic cells, as shown in Fig. S2A. We extended our observation on a wide range of hematopoietic malignant cells and confirmed that the presence of bacteria in culture can abrogate the anti-tumor activity of APO866 on all tested cell lines and primary hematopoietic malignant cells (Table S4 and Fig. 2).

Collectively, these results suggest that the efficacy of NAMPT inhibitors may be strongly reduced by bacteria and that such efficacy could possibly be restored by antibiotic treatment.

### Bacteria circumvent APO866-induced anti-leukemic activities by activating the Preiss-Handler pathway of NAD^+^ synthesis

**Fig. 3:**
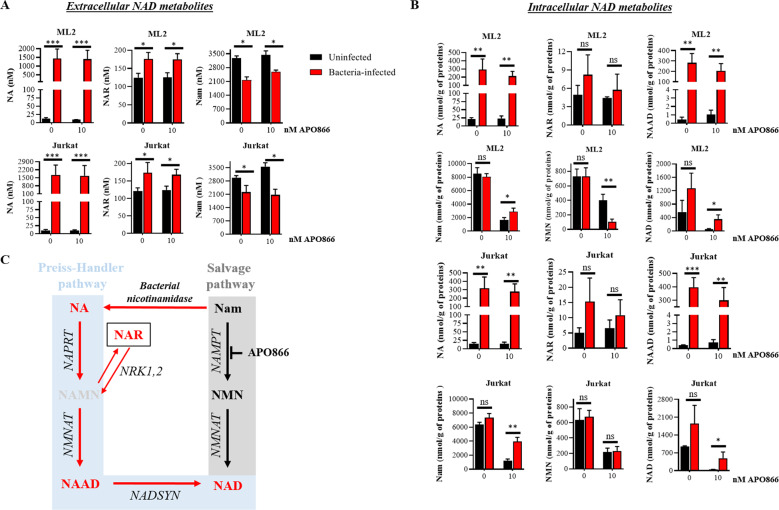
Bacteria circumvent APO866-induced anti-leukemic activities by activating the Preiss-Handler pathway to synthesize NAD^+^. Quantification of extracellular (**A**) or intracellular (**B**) NAD^+^ metabolites in cell-conditioned medium (or supernatant) or within cells from uninfected- or bacteria-infected ML-2 and Jurkat cells, using LC-MS/MS. **C** Summary of identified NAD^+^ metabolites: in red, metabolites significantly increased; black, no changes; and grey, metabolites, not detected (below the limit of detection). Data are derived from at least three independent experiments. Data are mean ± SD; *p < 0.05, **p < 0.01, ***p < 0.001, ns Not significant.

**Fig. 4:**
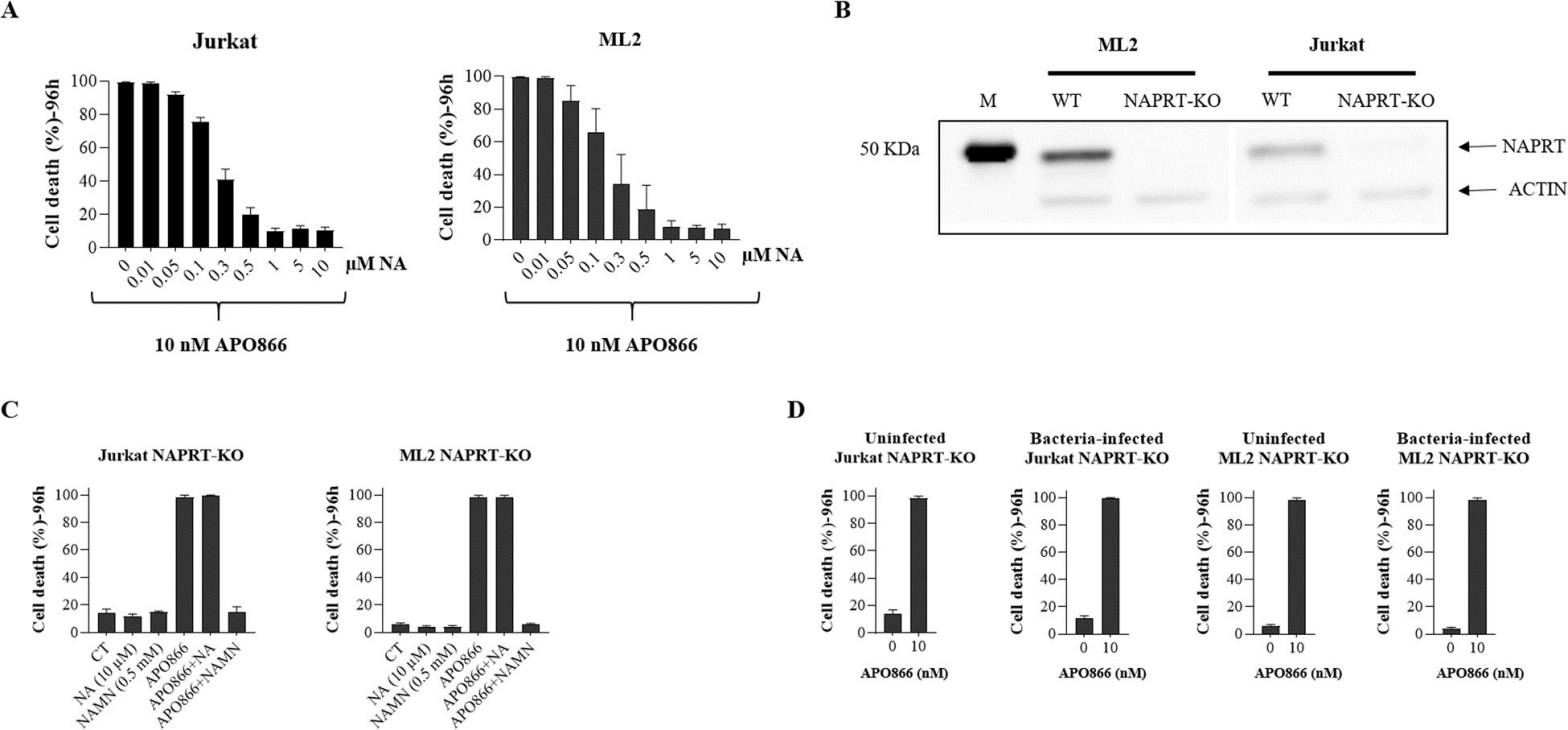
The abrogation of the anti-leukemic effect of APO866 in bacteria-infected leukemic cells requires the integrity of NAPRT. **A** ML-2 or Jurkat cells were incubated with or without various concentration of NA in presence of 10 nM APO866 for 96 h and cell death was assessed as described in Fig. 1. **B** NAPRT was knocked-out in wild type ML-2 and Jurkat cells using CRISPR/Cas9 technology; loss of expression was confirmed by Western blotting. **C** Jurkat NAPRT-KO and ML2 NAPRT-KO cells were pre-incubated with or without NA (10 µM) or NAMN (0.5 mM) before exposure to 10 nM APO866 for 96 h and cell death was assessed as described in Fig. 1. **D** Uninfected and bacteria-infected Jurkat/ML2 NAPRT-KO cells were treated without or with 10 nM APO866 and cell death monitored as described in Fig. 1. Data are derived from at least three independent experiments, and they are shown as mean ± SD.

**Fig. 5:**
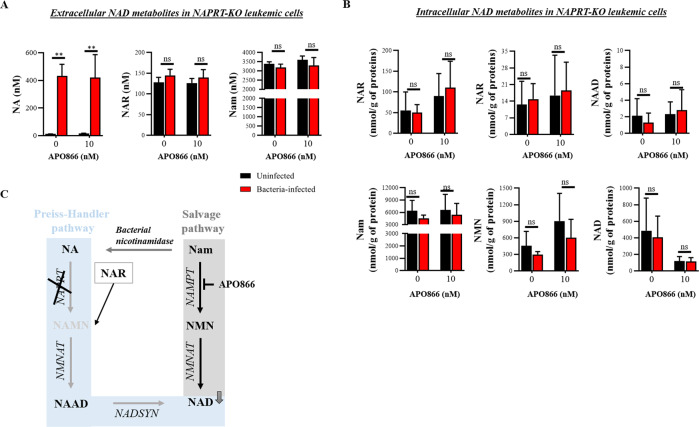
Bacteria-infected NAPRT-KO leukemic cells do not lead to activation of the Preiss-Handler pathway despite the significant increase of NA in the conditioned medium. Quantification of extracellular (**A**) or intracellular (**B**) NAD^+^ metabolites in cell-conditioned medium or within cells from uninfected or bacteria-infected NAPRT-KO ML-2 cells, using LC-MS/MS. **C** Summary of identified NAD^+^ metabolites: in red, metabolites significantly increased; black, no changes; and grey, metabolites not detected (below the limit of detection). Data are derived from at least three independent experiments. Data are mean ± SD, **p < 0.01, ns not significant.

Bacteria possess NMASE, which converts NAM to NA that serves as an NAD^+^ precursor for leukemic cells to synthesize NAD^+^ via the Preiss-Handler pathway. The resulting NAD^+^ production may therefore provide a mechanism to bypass the anti-cancer effects of NAMPT inhibition. To test this hypothesis, we measured by LC-MS/MS the content of NAD^+^ metabolites in conditioned medium (CM) from uninfected and from Mycoplasma-infected leukemia cells, in the absence or presence of 10 nM APO866. The CM from uninfected leukemic cells contained mainly NAM and just faint traces of NA (Fig. 3A). In the CM from Mycoplasma-infected leukemia cells, NAM was converted into NA, as evidenced by a significant increase in NA that correlated with a corresponding decrease in NAM. We next evaluated whether the presence of NA in the CM was followed by activation of the Preiss-Handler pathway for NAD^+^ production in leukemia cells. As shown in Fig. 3B and summarized in Fig. 3C, in comparison with uninfected leukemia cells, Mycoplasma-infected cells displayed a significant increase in NAD^+^ intermediate metabolites that are involved in NAD^+^ synthesis from NA. Consistent with our observation, Fig. 4A shows that supplementation with NA could abrogate the anti-tumor activity of APO866 and that less than 1 µM of NA is sufficient to protect cells against APO866-induced cytotoxicity. To demonstrate further that bacteria protect leukemic cells via the Preiss-Handler NAD^+^ production pathway, we generated leukemic cells in which NAPRT is knocked-out (KO) (Fig. 4B). As shown in Fig. 4C, in these NAPRT-KO cells, NA failed to prevent APO866-induced cell death, but the downstream product of NAPRT, nicotinic acid mononucleotide (NAMN), fully reversed APO866-mediated cell killing. In agreement with this observation, NAPRT-KO leukemic cells were highly sensitive to APO866 treatment despite the presence of Mycoplasma in cell culture (Fig. 4D). Finally, we measured the NAD^+^ metabolites in the CM from uninfected and Mycoplasma-infected NAPRT-KO leukemic cells treated with APO866. As expected, in these cells the presence of bacteria increased NA in the CM (Fig. 5A) but failed to increase intracellular NAD^+^ metabolites involved in the Preiss-Handler pathway (Fig. 5A–C). In agreement with the above-mentioned data, Mycoplasma-infected leukemic cells displayed an increased NMASE activity compared to uninfected ones (Fig. S2B). To extend this finding to any organism that possesses NMASE, we used unrelated microorganisms possessing the latter enzyme, namely *Escherichia coli* or *Saccharomyces cerevisiae*, to infect leukemic cells that were next incubated with or without APO866. Figure 2B shows that both *E. coli* and *S. cerevisiae* fully abrogated the APO866-induced cell death in untransfected but not in NAPRT-KO leukemic cells. To demonstrate that bacteria confer resistance to APO866 treatment, ML-2 cells exposed to different *E. coli* quantities (inocula), were treated without or with APO866 and cell death monitored. As expected, Fig. 2C shows that the protective effect to APO866 treatment correlated with bacterial copy number in culture. Of great interest, hematopoietic malignant cells supplemented with filtered (through a PVDF filter 0.1 µM) CM from either *E. coli* or *S. cerevisiae*-infected leukemic cell culture abolished APO866 cytotoxic activity (Fig. S2C), indicating that both microorganisms produce a soluble factor that could blunt the anti-tumor activity of NAMPT inhibitor.

Cancer stem cells (CSC) play a major role in drug resistance (or relapse) and are also known to significantly affect cancer therapy [34]. We examined whether CSC could be involved in the resistance to APO866 treatment. To this end, we examined the major CSC properties, such as stem cell markers (CD34, CD117, and CD123) expression and quiescence status of uninfected vs bacteria-infected leukemic cells. No stem cell markers were found on both uninfected and bacteria-infected leukemic cells (Fig. S3A) and there was no difference in terms of quiescent status between uninfected vs bacteria-infected leukemic cells (Fig. S3B). The results strongly suggest that CSC are not involved in APO866 resistance in our experimental model.

Altogether, the data suggest that whenever NAM is available, any NA-producing microorganisms such as various bacteria species (for instance gut microbiota), may strongly reduce the efficacy of NAMPT inhibitors by activating NAD^+^ production through the Preiss-Handler pathway in cancer cells. Silencing NAPRT in tumor cells could restore the therapeutic efficacy of NAMPT inhibitors even in the presence of bacteria and NAM.

### Tumor metabolic environment and intestinal bacteria inhibit the in vivo efficacy of APO866 in xenograft model of human leukemia

**Fig. 6:**
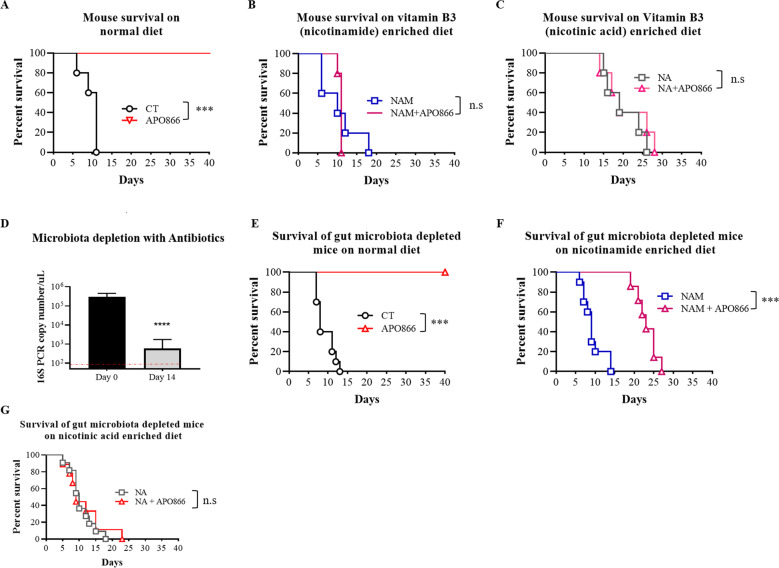
Tumor metabolic environment and intestinal bacteria severely hamper the in vivo efficacy of APO866 in xenograft model of human leukemia. In vivo efficacy of APO866 on SCID mice with intact (**A**–**C**; *n* = 5) or depleted intestinal bacteria (**E**–**G**; *n* ≥ 7), xenografted with ML-2 cells and fed with normal diet (**A**, **E**), enriched diet with NAM (**B**, **F**), or NA (**C**, **G**). 6- to 8-week-old C.B.-17 SCID mice were fed with different diets either poor in vitamin B3 (normal diet) or enriched in vitamin B3 (NAM or NA) for two weeks and subsequently treated with or without a cocktail of antibiotics to deplete intestinal microbiota before being transplanted subcutaneously with ML-2 cells. Once tumor sizes of 100–150 mm^3^ were reached, mice were randomized into a control group (black line) and a treated group (colored line). APO866 administration and tumor volume assessment were conducted as described in “Methods” section. All animals were monitored daily, and the study was ended when tumor volume reached around 1000 mm^3^. ***P < 0.001. **D** 16S rRNA PCR analysis on murine stool samples before and after antibiotic cocktail treatment using universal bacterial primers. Data are mean ± SD, *n* ≥ 9. Dot line indicates lower limit detection for PCR.

To examine the influence of the tumor environment on the anti-cancer activity of APO866, mice were fed with a diet that was poor in vitamin B3 (a standard diet contains 130 mg/kg of food NA), or with diets that were either enriched in NAM (1 g/kg of food) or in NA (1 g/kg of food) for two weeks before being grafted subcutaneously with ML-2 cells. In mice receiving the standard diet, treatment with APO866 cleared tumor cells and resulted in 100% survival for more than 5 weeks of observation, whereas all mice from the untreated group died within 2 weeks with tumors (Fig. 6A). In contrast, feeding mice with diet enriched in either NAM or NA abrogated the therapeutic efficacy of APO866 (Fig. 6B, C), strongly suggesting that the nutritional content of vitamin B3 should be considered to optimize the efficacy of NAMPT inhibitors. We next evaluated whether the intestinal microbiota could affect the efficacy of NAMPT inhibitors in leukemia treatment using the above-described in vivo xenograft model of human AML. Mice were fed with different diets (as described above) and were treated with a cocktail of antibiotics that was recently reported to be efficient at depleting intestinal bacteria [29]. Microbiota depletion was assessed weekly by analyzing bacterial content in stools, using universal 16S rRNA bacterial primers. As reported in Fig. 6D, antibiotic treatment decreased bacterial PCR copy number by 2 logs in comparison to stools collected before initiation of antibiotic treatment. As shown in Fig. 6E, in the control group (standard diet and without APO866 treatment), all mice died within 2 weeks post-transplantation, with a median survival of 8 days (95% confidence interval [3.8–60.5]). In the group of microbiota-depleted mice fed with the same standard diet, APO866 exerted a strong therapeutic effect, with 100% of mice surviving and remaining disease-free for the 40-day duration of the study. When antibiotic-treated mice were put on a NAM-enriched diet, administration of APO866 prevented tumor development and significantly prolonged mouse survival, with a median survival of 23 days (95% confidence interval [0.9 to 6.7]) compared with control which presented a median survival of 9 days (95% confidence interval [0.15–1.02]) (Fig. 6F). The result shows that NAM-enriched diet cannot fully abrogate APO866 anti-tumor effect when microbiota is depleted, hampering any conversion of NAM to NA to bypass NAMPT inhibition in tumors. In contrast, feeding mice with a NA-enriched diet completely abrogated the therapeutic efficacy of APO866, despite the depletion of gut microbiota with antibiotics (Fig. 6G). Antibiotic treatment per se had no effect on tumor growth. As in our previous studies [30, 32, 35–37], the chosen dose of APO866 (15 mg/kg) was well tolerated as demonstrated by no premature deaths and by the survival of all mice treated with APO866 throughout the 40 days of observation, without signs of toxicity including loss of body weight, lethargy or rough coat.

Collectively, these data underscore that the tumor metabolic environment as well as gut microbiota can interfere with the therapeutic efficacy of a NAMPT inhibitor.

## Discussion

Here we show that the amount of NAM or NA contained in food and of bacteria from the gut microbiota can severely modulate the anti-leukemia activity of a NAMPT inhibitor. Numerous bacteria species, including those found in gut microbiota, contain *PncA* [38], enabling them to convert NAM into NA, which in turn activates an alternative route of NAD^+^ biosynthesis, “the Preiss-Handler pathway”. This circumvents the NAD^+^ depletion that would normally be induced by the blockade of the NAD^+^ salvage pathway by NAMPT inhibitors. Accordingly, as an example, CM from Mycoplasma-infected cells displays high levels of NA that correlate with a decrease of NAM. Furthermore, supplementation with filtered CM from bacteria-infected cells or exogenous NA protects from APO866-induced tumor cell cytotoxicity both in vitro and in vivo. APO866 significantly depletes intracellular NAD^+^ content in both uninfected and bacteria-infected malignant hematopoietic cells but the residual NAD^+^ synthesized from NA is sufficient to prevent reactive oxygen species (ROS, Fig. S4A, S4B) production, preserve mitochondrial integrity (Fig. S5A), blunt the ATP depletion (Fig. S6) and thus protect leukemic/lymphoma cells from APO866-induced cell death (Fig. S5B). We provide evidence using xenograft model of human leukemia that gut microbiota can protect tumor cells from APO866-induced cell death if the diet is enriched in NAM. Importantly, our data strongly suggest that silencing/inhibition of NAPRT or antibiotic therapy could restore the anti-leukemia efficacy of APO866 despite the presence of bacteria.

The loss of the anti-tumor activity of NAMPT inhibitors in presence of bacteria supports the recent study by Shats et al. [24] reporting that bacteria can boost the mammalian host NAD^+^ and thereby confer resistance to NAMPT inhibitors in an in vivo setting of colon cancer. Here, we provide for the first time in vivo evidence that gut bacteria, despite being hosted at distant sites from a tumor, can protect leukemia/lymphoma cells from NAMPT inhibitor-induced death. This finding could be explained by the fact that bacteria produce a soluble factor (Fig. S2C) that circulate through the body and thus, confers resistance at distant sites. NMASE, which is present in many bacteria species [38–40], converts NAM to NA whenever NAM is made available to such bacteria, providing an alternative NAD^+^ precursor for cancer cells. Any therapeutic strategy aiming to block only one route of NAD^+^ biosynthesis should therefore be expected to fail in tumor cells in which the enzymatic apparatus of both NAD^+^ production routes (via NAMPT and via NAPRT) is expressed. Specifically, our data strongly suggest that two conditions are required for gut bacteria to protect tumor cells from APO866-induced cell death: 1) the expression of NAPRT and 2) the availability of sufficient amounts of NAM to bacteria. We demonstrate the possibility of reversing bacterial protection of leukemia cells by optimizing the NAD^+^-lowering therapy via two alternative strategies: i) simultaneously inhibiting both NAMPT and NAPRT or ii) combining NAMPT inhibition with oral antibiotic therapy aiming at depleting gut microbiota. Both approaches warrant being tested in clinical studies.

Our data indirectly support the concept that sensitizing cancer cells to NAMPT inhibitors in humans may need to combine them with additional inhibitor(s). We recently showed that inhibiting NAPRT could prove useful for sensitizing several cancer cells to NAMPT inhibitors [36]. Furthermore, these inhibitors could be specifically targeted to tumoral cells to prevent toxicity due to profound NAD^+^ depletion in healthy cells. Of note, NAMPT inhibition can be combined with inhibitors of metabolic or signaling pathways that act synergistically to kill tumoral cells. This was exemplified by Zhang et al. [41], who provided evidence that inhibition of two histone deacetylases, HDAC8 and SIRT6, enhances the anti-leukemic effect of NAMPT inhibitor in AML with minimal toxicity to healthy cells.

Although in our context, bacteria confer resistance to NAMPT inhibitors mainly via modulation of NAD^+^ metabolites, there is growing evidence indicating that host commensal microbiota can also modulate the efficacy of cancer therapy via other mechanisms that include immunomodulation, activation of autophagy, translocation, and drug metabolism [42–45]. We cannot firmly rule out the possibility that the resistance to NAMPT inhibitors observed in the present study could also reflect, at least in part, such additional mechanisms and thus, further experiments are warranted to elucidate this issue.

In conclusion, we demonstrate that a NAM-rich diet, through its take-up by the gut microbiota, can confer resistance to APO866, a very potent NAMPT inhibitor. Lowering specific NAD^+^ precursors in the diet, silencing or inhibiting NAPRT in cancer cells or depleting the gut microbiota using antibiotics are all potential avenues for optimizing the activity of NAMPT inhibitors in hematological malignancies.

## Supplementary information

Suppl. Files

Original Data File

reproducibility checklist

## Data Availability

The datasets generated or analyzed during the current study are available from the corresponding author on reasonable request.
