# Supplementary material for: Gut microbiota severely hampers the efficacy of NAD-lowering therapy in leukemia
Source: Cell Death Dis. 2022 Apr 8;13(4):320. doi: 10.1038/s41419-022-04763-3 (PMC8993809; doi:10.1038/s41419-022-04763-3)
Supplement: Supplementary file 1 — Suppl. Files [file 41419_2022_4763_MOESM1_ESM.pdf]

Table S1. Cell line description

Disease diagnosis	Cell line	Provider	Catalogue no.
Acute Myeloid Leukemia (AML)	HEL (M6)	DSMZ	ACC 011
	MV4-11 (M5)	DSMZ	ACC 102
	THP-1	ATCC	TIB-202
	SKM-1 (M5)	DSMZ	ACC 547
	NOMO-1 (M5)	DSMZ	ACC 542
	ML-2 (M5)	DSMZ	ACC 015
	NB-4 (M3)	DSMZ	ACC 207
	HL-60 (M2)	ATCC	CCL-240
	Kasumi (M2)	DSMZ	ACC 220
T-Acute Lymphoblastic Leukemia (T-ALL)	Jurkat	ATCC	TIB-152
	CCRF-CEM	DSMZ	ACC 240
	MOLT-4	DSMZ	ACC 362
B-Chronic Lymphoblastic Leukemia (B-CLL)	Mec-1	DSMZ	ACC 497
Burkitt Lymphoma	Ramos	DSMZ	ACC 603
Multiple Myeloma (MM)	RPMI-8226	ATCC	CCL-155
	MOLP-8	DSMZ	ACC 569

ATCC: American Type Culture Collection; DSMZ: German Collection of Microorganisms and Cell cultures

Table S2. Primary cells from patients

Patient diagnosis	Sample no	Place
Acute Myeloid Leukemia (AML)	AML #1	CHUV
	AML#2	CHUV
Mantle Cell Lymphoma (MCL)	MCL	CHUV
B-Chronic Lymphoblastic Leukemia (B-CLL)	B-CLL #1	CHUV
	B-CLL #2	CHUV

CHUV: Centre Hospitalier Universitaire Vaudois

Table S3. Leukemic cells are infected with Mycoplasma arginini

Cell line	Species	Myoplasma species	Conclusion
Uninfected ML2 WT	Human		clean
Uninfected Jurkat WT	Human		clean
Mycoplasma-infected ML2	Human	*Mycoplasma arginini*	Contaminated
Mycoplasma-infected Jurkat	Human	*Mycoplasma arginini*	Contaminated

Table S4. Bacteria abrogate the anti-leukemic effects of APO866 in different primary leukemic cells

	**Cell death (mean +/-SD); 10nM APO866, 96h**	
**Disease fiagnosis/ cell line**	**Uninfected**	**Bacteria-infected**
Acute Myeloid Leukemia (AML)		
MV4-11	98.5 +/- 1.2	4.6 +/- 0.3
NOMO-1	98.2 +/- 1.1	4 +/- 0.1
SKM-1	98.1 +/- 0.05	4.3 +/- 0.3
THP-1	98.7 +/- 0.9	4.1 +/- 0.4
NB-4	96.8 +/- 0.5	3.8 +/- 0.5
HL-60	87.7 +/- 0.7	3.8 +/- 0.5
Acute lymphoblastic leukemia		
CCRF-CEM	82.6 +/- 4.6	14.4 +/- 0.3
MOLT-4	87.7 +/- 0.6	13.6 +/- 0.7
B-Chronic Lymphocytic Leukemia (B-CLL)		
Mec-1	60.4 +/- 1.3	9.1 +/- 0.7
Burkitt lymphoma		
Ramos	93 +/- 3	11.1 +/- 1.5
Multiple myeloma		
RPMI-8226	80.4 +/- 1.3	9.5 +/- 0.8
MOLP-8	95 +/- 1.2	9 +/- 0.2

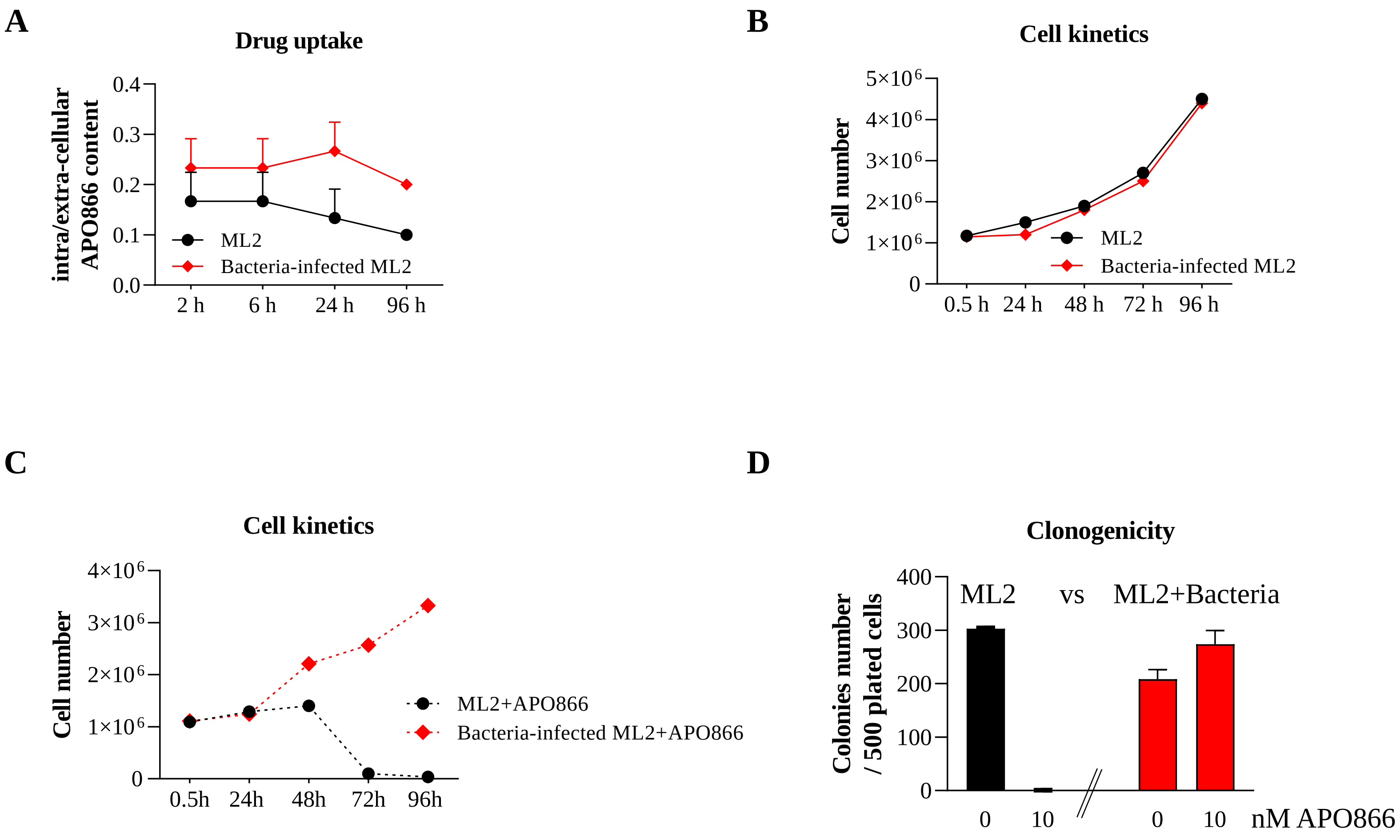

Figure S1. Bacteria protect leukemic cells from the anti-tumor activities of APO866 without affecting their drug uptake, capacity to proliferate, and propensy to form colonies.

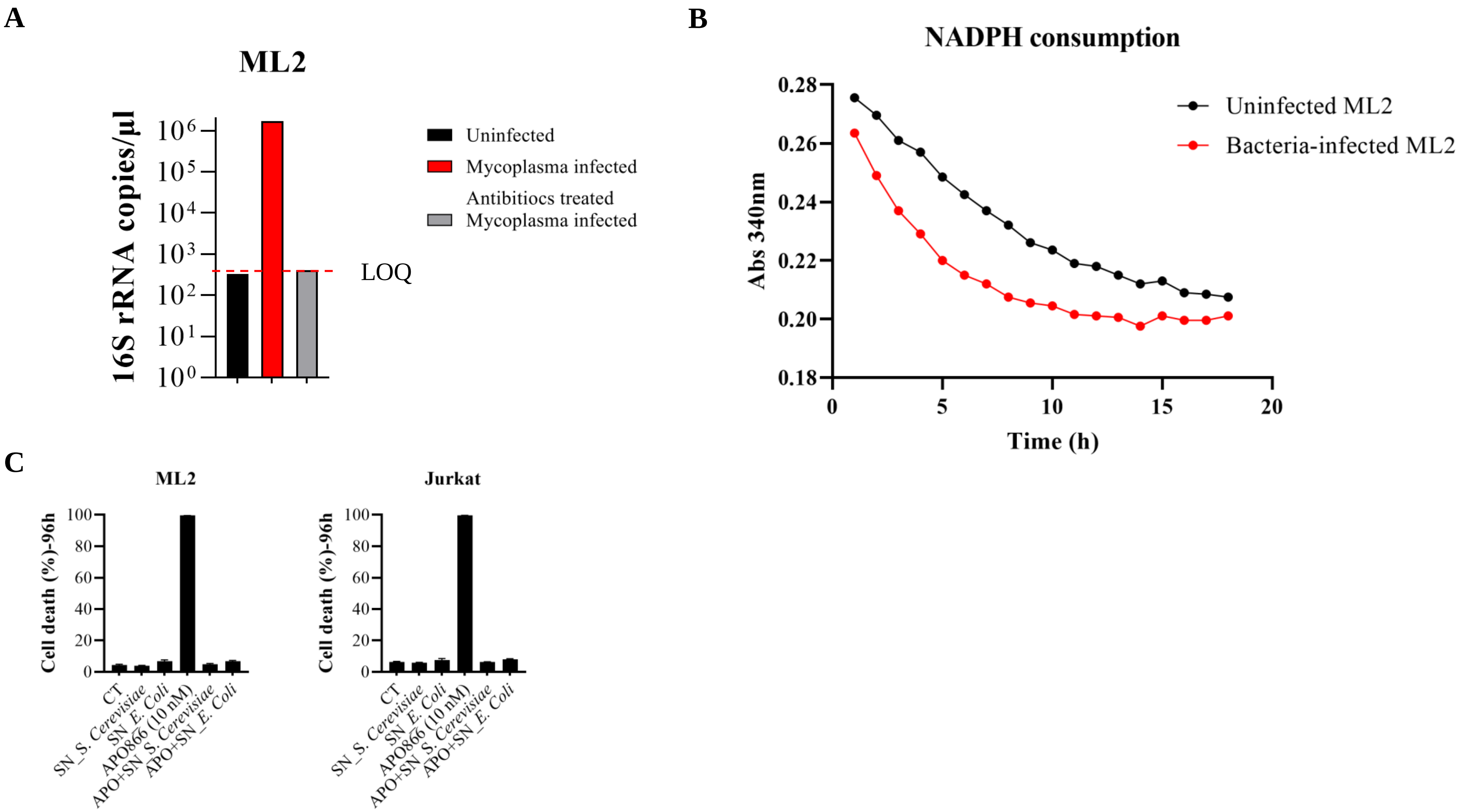

Figure S2. Antibiotic (BM cyclin) treatments eliminate Mycoplasma from infected-leukemic cells (A). Bacteria-infected cells display an increased nicotinamidase activity compared to uninfected ones (B) . Filtered supernant from *E. coli* infected leukemic cells confers resistance to APO866 (C).

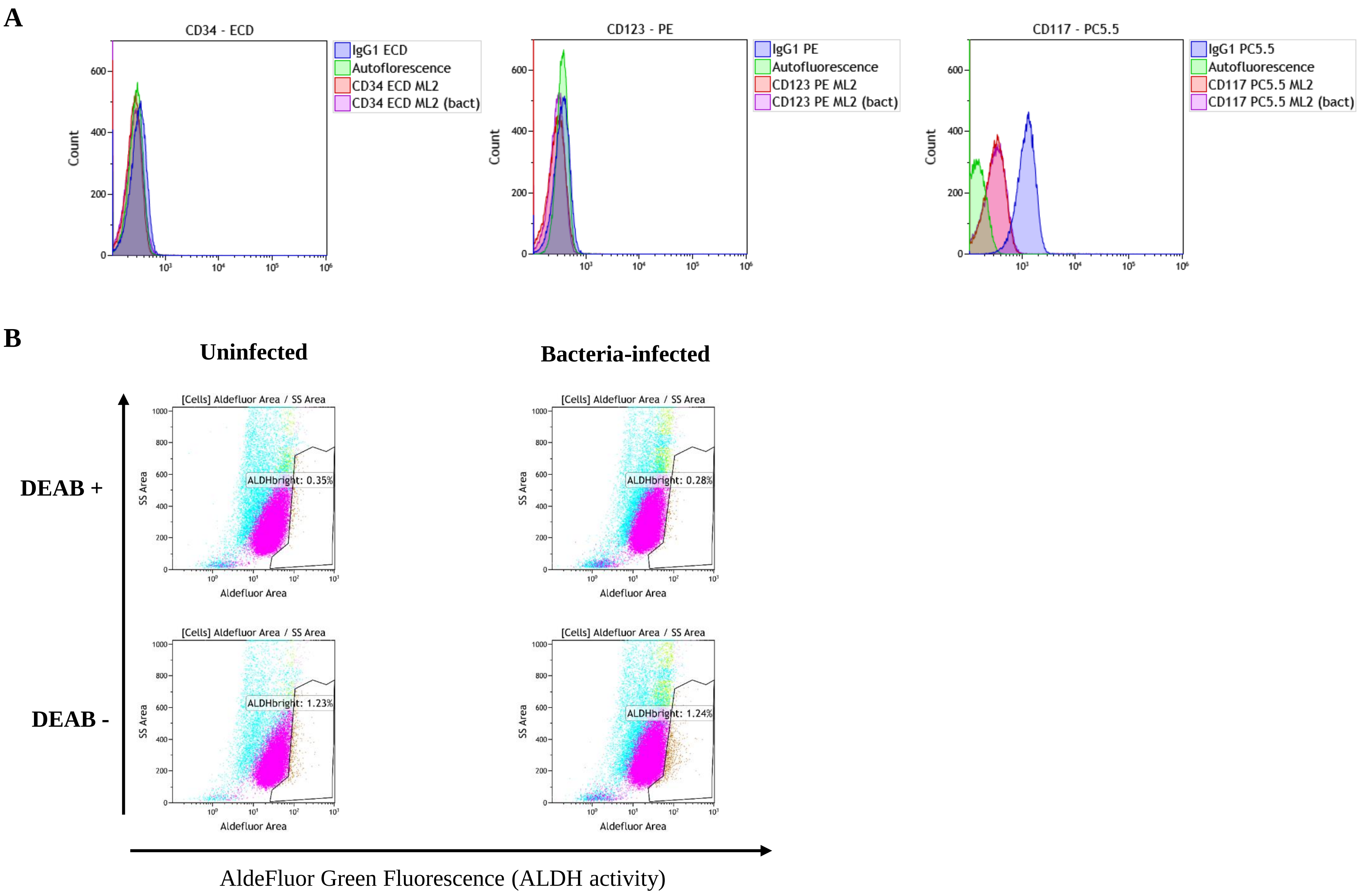

Figure S3. Uninfected and bactera-infected leukemic cells do not present in stem cell markers expression (A) as well as in quiescent status evaluated by AldeFluor Assay (Stem Cell techonologies) (B).

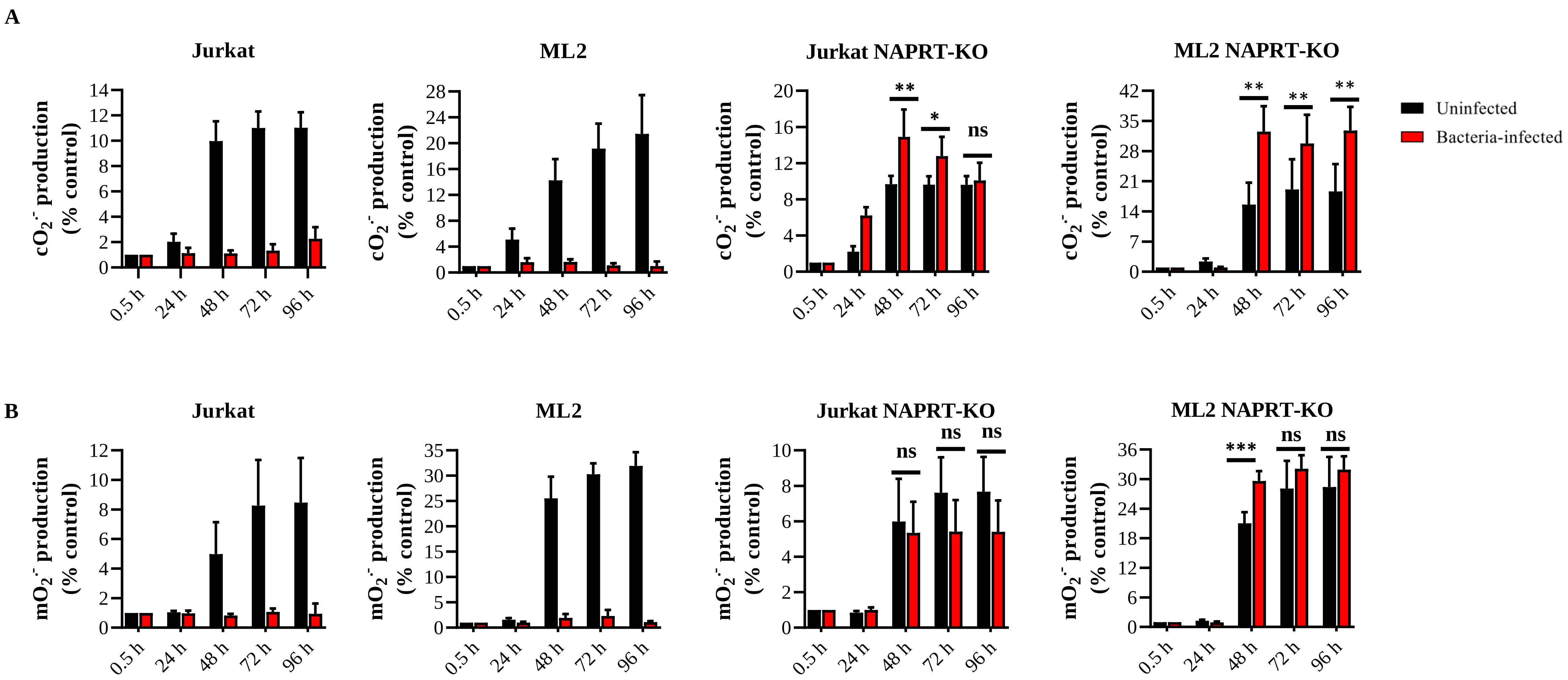

Figure S4. Bacteria blunt APO866-induced oxidative stress in WT but not in NAPRT-KO leukemic cells.

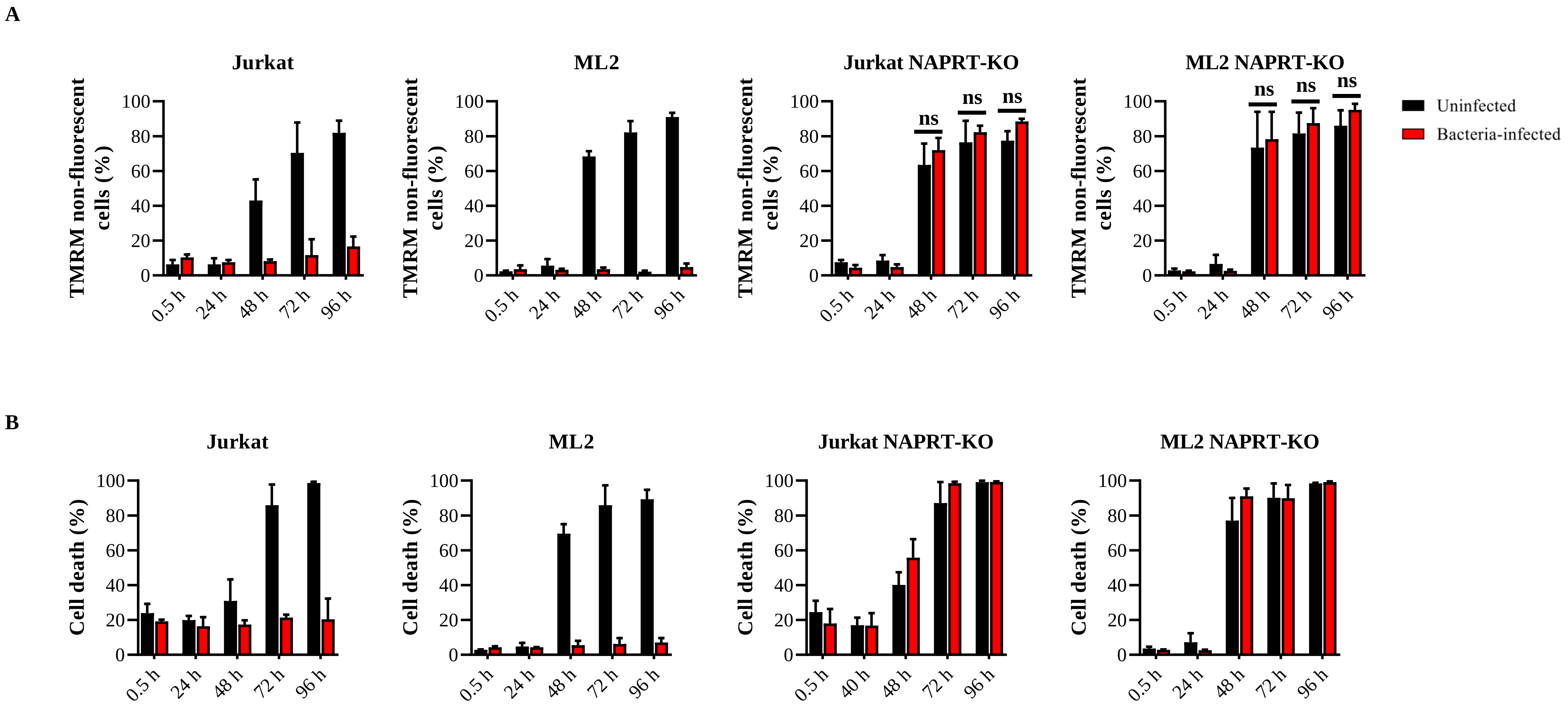

Figure S5. Bacteria abrogate loss of mitochondrial membrane potential and cell death induced by APO866 in WT but not in NAPRT-KO leukemic cells

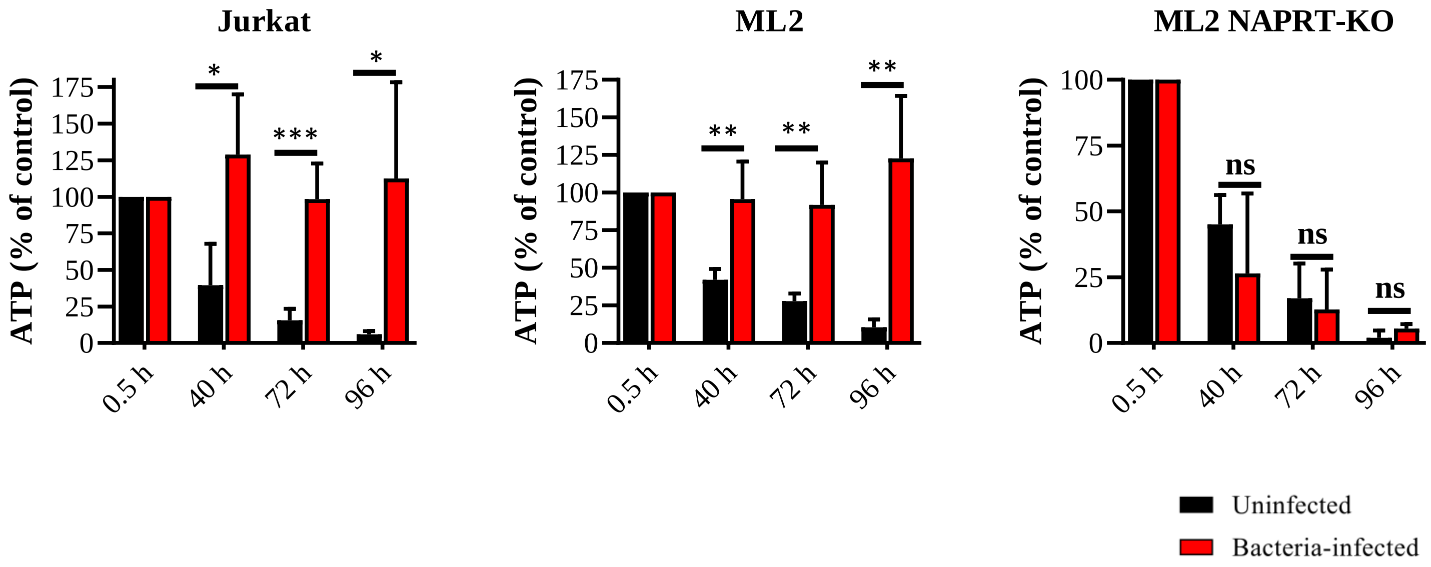

Figure S6. Bacteria prevent ATP depletion induced by APO866 in WT but not in NAPRT-KO leukemic cells
