# Supplementary figures and images for: Gut microbiota severely hampers the efficacy of NAD-lowering therapy in leukemia
Source: Cell Death Dis. 2022 Apr 8;13(4):320. doi: 10.1038/s41419-022-04763-3 (PMC8993809; doi:10.1038/s41419-022-04763-3)

Western Blot original images

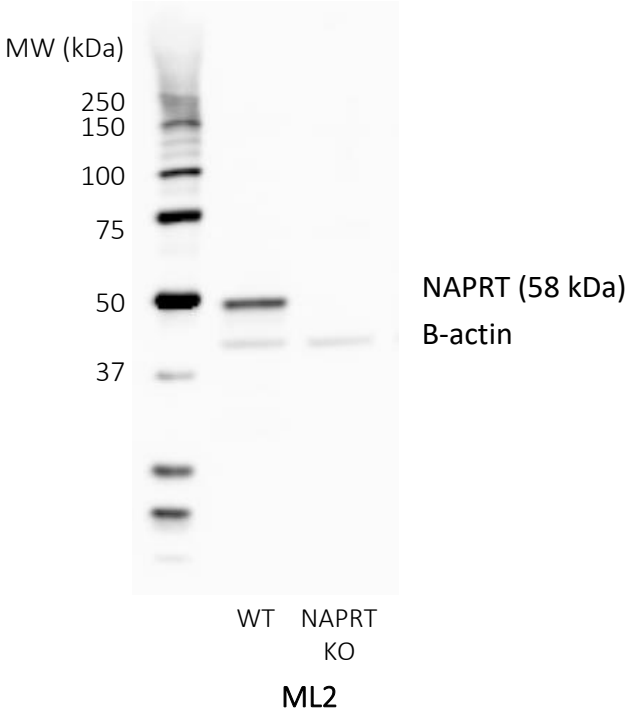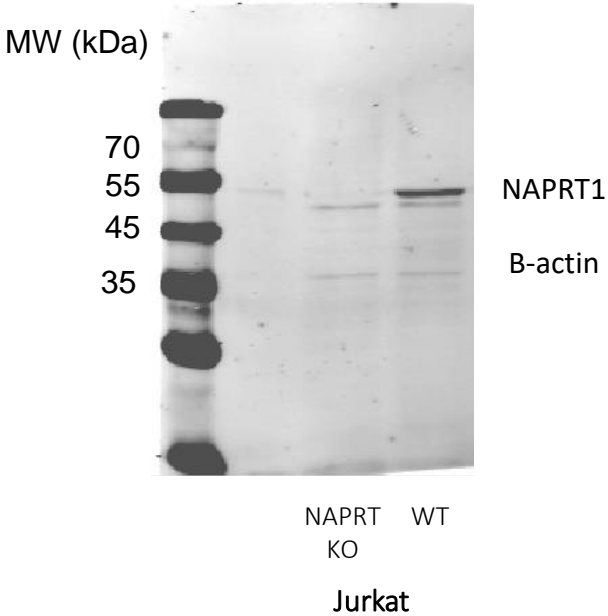

Supplement: Supplementary file 2 — Original Data File [file 41419_2022_4763_MOESM2_ESM.pdf]
